# Real-world experience with deucravacitinib in psoriatic patients: the Chilean perspective^؆^

**DOI:** 10.1016/j.abd.2025.501264

**Published:** 2026-01-05

**Authors:** Fernando Valenzuela Ahumada, Jorge Contreras Aguilera, Felipe Alcayaga de la Ribera, Raúl Cabrera Moraga, Cristóbal Lecaros Cornejo

**Affiliations:** a Department of Dermatology, Universidad de Chile, Santiago, Chile; b Department of Dermatology, Universidad de los Andes, Santiago, Chile; c Department of Dermatology, Universidad del Desarrollo, Santiago, Chile

Dear Editor,

We present the findings of a multicentre observational study evaluating the efficacy and safety of deucravacitinib (Sotyktu®) in a cohort of Chilean patients with moderate-to-severe plaque psoriasis. Psoriasis affects approximately 1.1% of the Chilean population, with an incidence of 22 per 100,000 inhabitants, highlighting the need to document the real-world performance of novel therapies.

From July 2023 to August 2024, 24 adults were recruited from four dermatology centres in Santiago. Deucravacitinib was prescribed at 6 mg orally once daily. Disease severity was assessed with the Psoriasis Area and Severity Index (PASI) and Body Surface Area (BSA), and quality of life with the Dermatology Life Quality Index (DLQI). Evaluations were performed at baseline and at 3-, 6-, and 12-months. Adverse Events (AEs) were summarized descriptively with R (v 4.3.2).

Seventeen participants were male; the mean ± SD age was 38.5 ± 9.3 years, and the mean disease duration 16.1 ± 11.99 years. Fifteen patients had at least one comorbidity, and most had previously received methotrexate and/or biologics. Baseline mean PASI was 18 ± 12.4, mean BSA 15 ± 0.13 %, and mean DLQI (n = 16) 24 ± 3.75 (Table 1).

**Table 1:** Baseline characteristics of patients with psoriasis treated with Deucravacitinib (n = 24).

**i. Demographic Data**	
Male sex, n (%)	17 (70.83%)
Age (years)^a^	38.58 ± 10.56
Age of Psoriasis Onset (years)^a^	22.91 ± 14.25
Psoriasis duration (years)^a^	16.10 ± 11.99
**ii. Clinical indexes**	
Total PASI^a^	18.03 ± 12.44
BSA (%)^a^	15 ± 0.13
DLQI^a^	24.06 ± 3.75
**iii. Comorbidities**	
**Total comorbidities (n)**	15
Obesity	2
Hypertension	3
Type 2 Diabetes Mellitus	3
Psoriatic arthritis	1
Mood disorder	5
Other	8
No comorbidities	9
**iv. Previous treatments**	
**Total previous treatments (n)**	24
Topical treatments	20
Methotrexate	12
Phototherapy	14
Anti-IL23	2
Anti-TNFα	1
Anti-IL17	1
No prior treatment	0

PASI, Psoriasis Area and Severity Index; DLQI, Dermatology Life Quality Index.

a Data presented as mean ± standard deviation.

At month 3 (n = 21), PASI-75, PASI-90 and PASI < 3 were achieved by 33.3%, 9.5% and 23.8% of patients, respectively, with DLQI falling to 7.3. At month 6 (n = 21), the corresponding proportions were 70.8%, 38.1% and 57.1%; at month 12 (n = 19), they were 78.9%, 31.6% and 78.9% (Fig. 1).

**Fig. 1:**
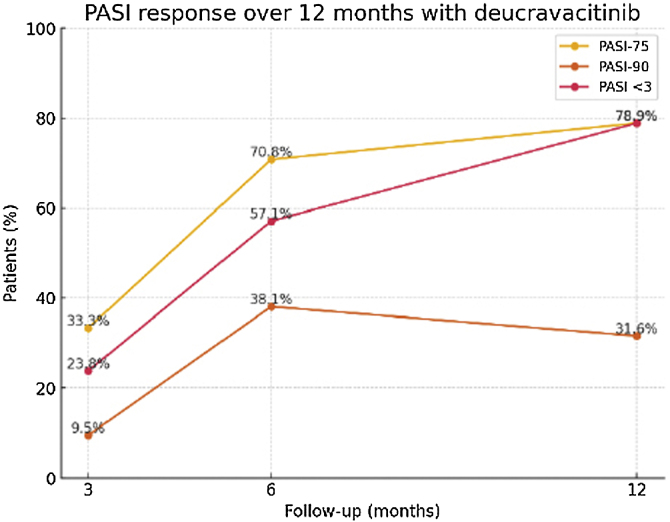
Evolution of PASI at months 3, 6 and 12 in patients with Deucravacitinib: 21 patients were in follow-up at 3 months and 6 months, 19 patients were in follow-up at 12 months.

Reported AEs were mild: acneiform eruption (n = 1), self-limiting upper-respiratory infections (n = 2), and headache (n = 1). One patient discontinued treatment owing to loss of efficacy.

Two-proportion *z*-tests showed significant improvements from month 3 to 6 for all outcomes (p < 0.05). No further significant change occurred between months 6 and 12, indicating a clinical plateau. Compared with month 3, PASI-75 (p = 0.0038) and PASI < 3 (p = 0.0005) remained significantly higher at month 12, whereas the increase in PASI-90, although numerically greater, did not reach conventional significance (p = 0.081).

Deucravacitinib delivered rapid and durable clinical benefit with a favorable safety profile in this real-world Chilean cohort, mirroring or exceeding the efficacy reported in pivotal trials.^1^, ^2^, ^3^ The superior responses observed may reflect the permitted use of concomitant topical therapy. Our results also align with a recent Japanese series, which reported PASI-75 in 78.3% and PASI-90 in 52.2% of patients at week 16.^4^ Continued assessment for 12 months is a notable strength of the present study.

These data support deucravacitinib as an effective first-line option for the management of moderate-to-severe psoriasis. Longer-term, larger-scale studies are warranted to refine its place in therapy within Latin-American and global populations.

## ORCID IDs

Fernando Valenzuela Ahumada: 0000-0003-1032-9347

Jorge Contreras Aguilera: 0009-0004-2307-3699

Felipe Alcayaga de la Ribera: 0009-0007-1994-7447

Raúl Cabrera Moraga: 0000-0002-0180-9130

Cristóbal Lecaros Cornejo: 0000-0002-8509-1188

Received 15 June 2025; accepted 26 August 2025

## Authors' contributions

Fernando Valenzuela Ahumada: Conceptualization, study design, data collection, data analysis, and manuscript drafting and revision.

Jorge Contreras Aguilera: Data collection, data analysis, and manuscript drafting and revision.

Felipe Alcayaga de la Ribera: Data collection, data analysis, and manuscript drafting and revision.

Raúl Cabrera Moraga: Data collection, manuscript revision

Cristóbal Lecaros Cornejo: Data collection, manuscript revision.

## Financial support

This study received no financial support.

## Research data availability

The entire dataset supporting the results of this study was published in this article.

## Conflicts of interest

Fernando Valenzuela has served as advisor and/or paid speaker for and/or participated in clinical trials sponsored by Pfizer Inc., AbbVie, Amgen, BMS, Janssen-Cilag, LEO, Lilly, Novartis, and Sanofi.
